# The Muse in Medicine

**Published:** 1883-02

**Authors:** 


The Muse in Medicine.—In the January number of the *Southern Practitioner* is a poem of twenty-six stanzas entitled "What is Life?" by T. O. Summers, A. M., M. D., who is Professor of Phisiology and Clinical Surgery in the Medical Department of the University of Tennessee. We quote the following:

Oh, Savant, tell us, what is life,
With all its wondrous springs?
Is it the rush, and whirl, and strife,
Which living always brings?

Thou child, so pure and innocent,
What answer dost thou give?
Is life but days of pleasure spent?
Is it but this to live?

Oh, son of toil, what sayest thou?
Is life but earning bread,
With drops of sweat upon thy brow,
And rest upon thy bed?

Answer, red-handed son of war,
In life what dost thou see?
Naught but the cannon-riven air
And shouts of victory?

Within the darkness of the womb,
Life's forces are begun,
And in the silence of the tomb
Its feverish strife is done.

